# A Theoretical Simulation of the Radiation Responses of Si, Ge, and Si/Ge Superlattice to Low-Energy Irradiation

**DOI:** 10.1186/s11671-018-2547-9

**Published:** 2018-05-02

**Authors:** Ming Jiang, Haiyan Xiao, Shuming Peng, Guixia Yang, Zijiang Liu, Liang Qiao, Xiaotao Zu

**Affiliations:** 1 School of Physics, University of Electronic Science and Technology of China, Chengdu, 610054 China; 2 Institute of Nuclear Physics and Chemistry, Chinese Academy of Engineering Physics, Mianyang, 621900 China; 3 Department of Physics, Lanzhou City University, Lanzhou, 730070 China

**Keywords:** Superlattice, Si and Ge, Radiation, Defect formation and migration

## Abstract

In this study, the low-energy radiation responses of Si, Ge, and Si/Ge superlattice are investigated by an ab initio molecular dynamics method and the origins of their different radiation behaviors are explored. It is found that the radiation resistance of the Ge atoms that are around the interface of Si/Ge superlattice is comparable to bulk Ge, whereas the Si atoms around the interface are more difficult to be displaced than the bulk Si, showing enhanced radiation tolerance as compared with the bulk Si. The mechanisms for defect generation in the bulk and superlattice structures show somewhat different character, and the associated defects in the superlattice are more complex. Defect formation and migration calculations show that in the superlattice structure, the point defects are more difficult to form and the vacancies are less mobile. The enhanced radiation tolerance of the Si/Ge superlattice will benefit for its applications as electronic and optoelectronic devices under radiation environment.

## Background

During the past decades, the Si/Ge superlattice (SL) has attracted much attention in semiconductor research due to its potential contribution to the development of new electronic and optoelectronic devices [1–6]. For example, the study of photoconductivity of Si/Ge SL is of remarkable importance for photodiodes as emitter and receiver for fast optical communication [5]. In its applications like the space electronic component, the microelectronic component, the solar cell and the space-based electronics [1, 4, 6], the optical and electronic properties of Si/Ge SL may be altered due to the bombardment of high-energy ions from space environment, resulting in performance degradation of the electronic devices. Therefore, it is necessary to investigate the radiation responses of this semiconductor material under extreme working conditions.

Recently, a lot of researchers have studied the radiation damage effects of Si/Ge superlattice [7–16]. Sobolev et al. investigated the influences of electron irradiation on the photoluminescence (PL) of Si/Ge SL containing monolayer of pure Ge, and enhanced radiation resistance of the SL structure was found as compared with bulk silicon [12]. Fonseca et al. irradiated the Si/Ge SL with embedded Ge quantum dots (QDs) employing the 2.0 MeV proton irradiation and found an extraordinary high radiation resistance of the QD-in-SL structure [13]. Similar results were obtained by Leitão et al., who reported that the Ge quantum wells (QWs) deposited on a diode structure containing a Si/Ge multilayer structure were more resistant to the proton irradiation as compared with the single Ge QWs [14]. As the promising thermoelectric materials, the thermoelectric characteristic of Si/Ge system may be also affected under the radiation environment [11, 15]. Zheng et al. irradiated the multiple periodic layers of Si_1 − *x*_Ge_*x*_/Si employing 5 MeV Si ions, and they found that the thermo-electric figure of merit increases with increasing Si ions fluencies [11]. The defects and structural disorder reduce the cross plane thermal conductivity by absorbing and dissipating phonon along the lattice, and the electronic density of states in the miniband of the QD structure increases the electrical conductivity and the Seebeck coefficient, which all contribute to the increase of figure of merit [11].

Theoretically, Sayed and Windl both investigated the atomic displacements of bulk Si employing the classical molecular dynamics (MD) method [17, 18]. They found that the threshold displacement energies (E_d_s) depend on the knock-on direction and the damaged states are mainly Frenkel pair (FP) defects [17, 18]. Caturla et al. studied the effects of ion mass and energy on the radiation damage of bulk Si employing the MD method [19]. They reported that the production of amorphization as well as isolated point defects and small clusters have a strong dependence on ion mass and a weak relationship to ion energy [19]. Holmström et al. calculated the E_d_s for germanium using the MD method and found that the stable defects are FP defects [20]. Shaw et al. applied an ab initio method to study the effects of antimony and germanium defects on the electronic structure of Si/Ge heterostructures and found that these defects interact with the Si/Ge interfaces, resulting in interface-related localized resonances and large local perturbations to the electronic structure [21]. Despite of these mentioned investigations, no theoretical simulations of dynamic process of radiation damage of Si/Ge SL have been reported in the literature thus far. There still lacks an atomic-level understanding of the micro-structural evolution and the underlying mechanism for defect generation in the semiconductor superlattices.

The ab initio molecular dynamics (AIMD) method has been demonstrated to be an important tool for shedding light on the radiation damage processes and has indeed been successful in simulating the recoil events of a series of semiconductor and ceramic materials [22–27]. As compared with the classical MD method, the interatomic potentials are obtained from electronic structure calculations rather than empirical fitting of experimental results. Consequently, a lot of physical parameters like E_d_s can be determined with ab initio accuracy. In this study, the AIMD method is employed to compare the response behaviors of bulk Si, Ge, and Si/Ge SL under low-energy irradiation. The threshold displacement energies have been determined, and the defect distribution and the pathway for defect generation have been provided. The possible origin for the discrepancy in radiation tolerance between bulk Si (Ge) and Si/Ge SL is also explored. The presented results provide a fundamental insight into the microscopic mechanism of displacement events in bulk Si, Ge, and Si/Ge SL and advance the understanding of the radiation responses of these materials under radiation environment.

## Methods

**Fig. 1:**
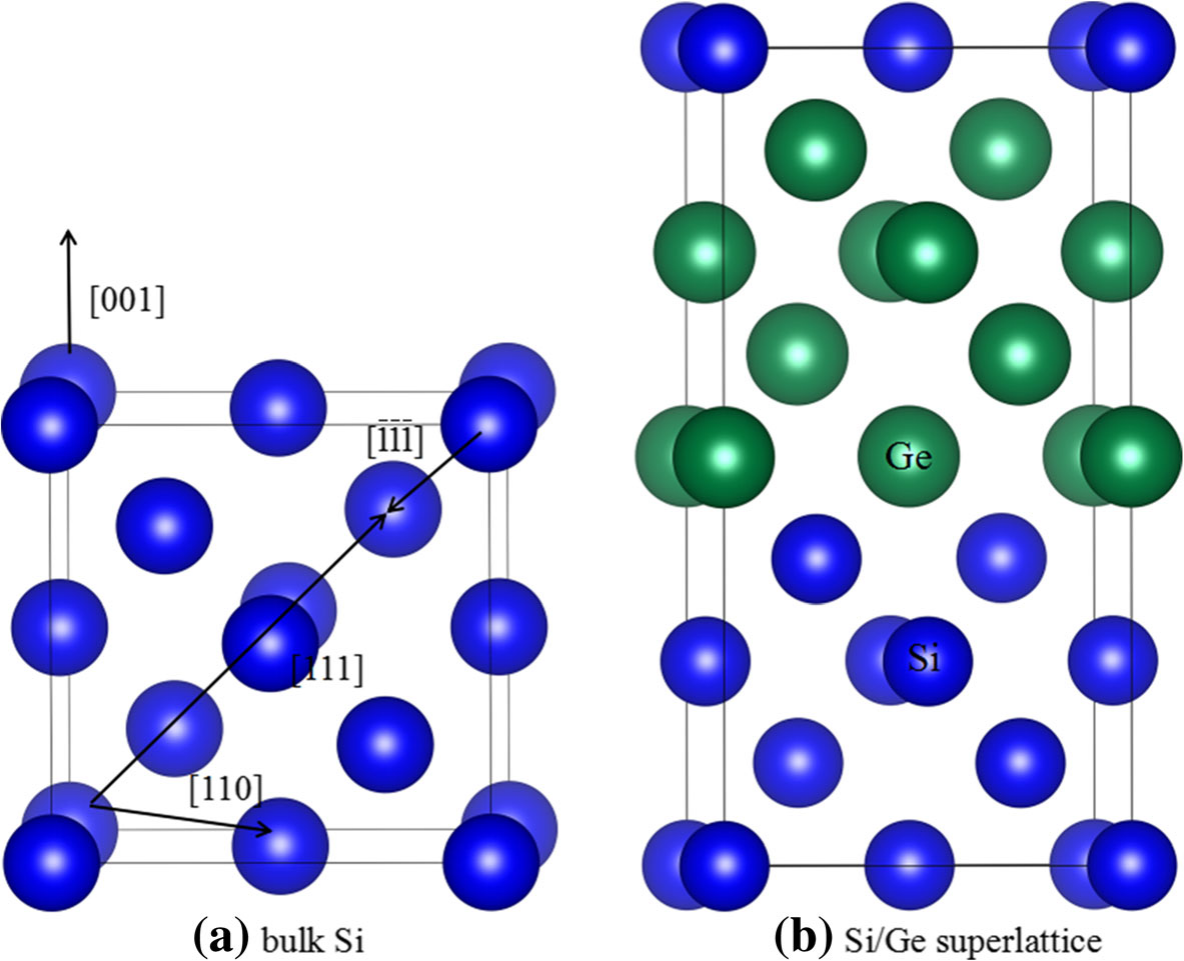
Schematic view of geometrical structures of **a** bulk Si and **b** Si/Ge superlattice. The blue and green spheres represent the Si and Ge atoms, respectively

The low-energy displacement events of bulk Si, Ge, and Si/Ge SL are simulated by the Spanish Initiative for Electronic Simulations with Thousands of Atoms (SIESTA) code. The norm-conserving Troullier-Matrins pseudopotentials [28] are employed to determine the interaction between ions and electrons, and the exchange-correlation potential is described by the local-density approximation (LDA) in Ceperly-Alder parameterization [29]. The valence wave functions are expanded by a basis set of localized atomic orbitals, and single-ζ basis sets plus polarization orbital (SZP) are employed, with a K-point sampling of 1 × 1 × 1 in the Brillouin zone and a cut-off energy of 60 Ry. In the present study, a Si_2_/Ge_2_ SL, which consists of two layers of Si alternating with two layers of Ge and totally 288 atoms, is considered. Figure 1 illustrates the geometrical configuration for bulk Si and Si/Ge SL. A specific atom is selected as the primary knock-on atom (PKA), and it is given a kinetic energy to initiate a recoil event. If the PKA returns to its original position at the end of the displacement event, the simulation is restarted at higher recoil energy with an energy increment of 5 eV. Once the PKA is permanently displaced from its lattice site, additional runs are preformed to improve the precision to 0.5 eV. For each atom type, four and five principal incidence directions are taken into account for bulk Si (Ge) and Si/Ge SL, respectively. The simulations are conducted with an NVE ensemble and the maximum duration of each run is 1.2 ps to avoid the instability of the system.

## Results and Discussion

### The Displacement Events in Bulk Silicon and Germanium

**Fig. 2:**
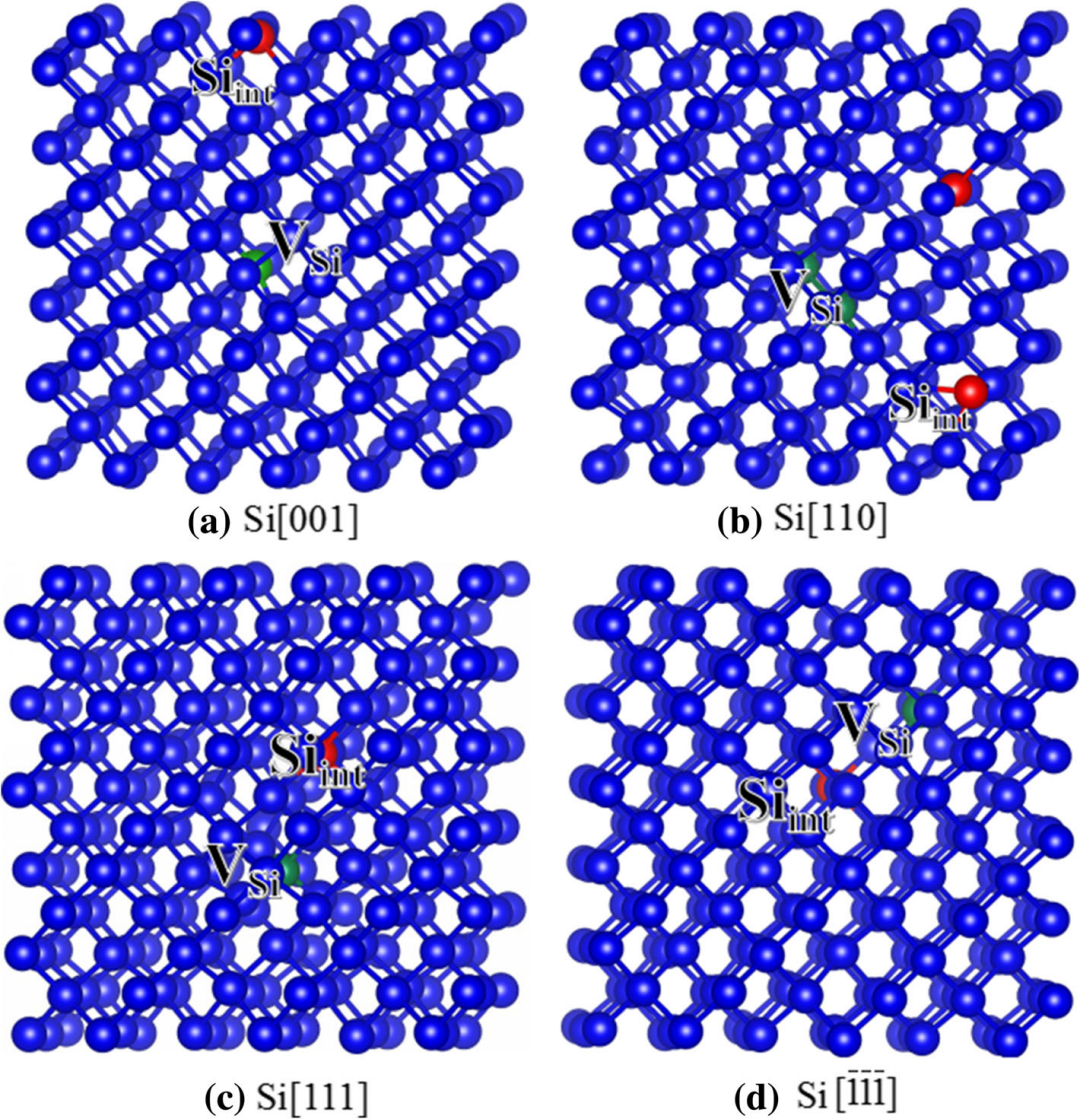
**a**–**d** Schematic view of geometrical structures of damage Si after recoil events. The green and red spheres represent the vacancy and interstitial defects, respectively. V_Si_: silicon vacancy; Si_int_: silicon interstitial

**Fig. 3:**
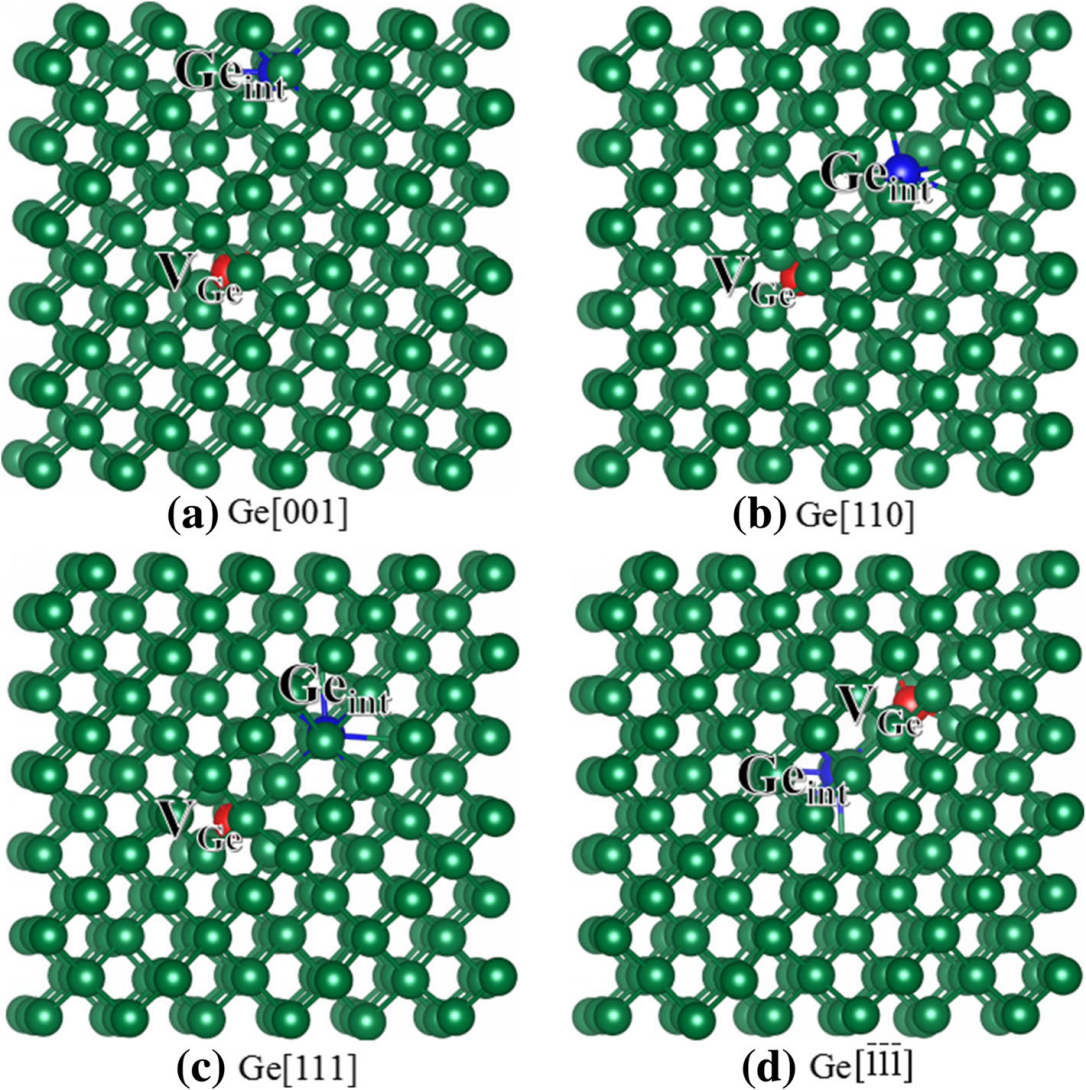
**a**–**d** Schematic view of geometrical structures of damage Ge after recoil events. The red and blue spheres represent the vacancy and interstitial defects, respectively. V_Ge_: germanium vacancy; Ge_int_: germanium interstitial

The lattice constant of bulk Si is determined to be 5.50 Å, which agrees well with the theoretical result of 5.48 Å [30] and the experimental result of 5.43 Å [31]. As compared with bulk Si, the lattice constant of bulk Ge is larger, i.e., 5.71 Å, which is consistent with the calculated result of 5.65 Å [30] and the experimental value of 5.77 Å [31]. Our calculated threshold displacement energies for bulk Si and Ge are summarized in Table 1, along with the associated defects after the displacement events. The configurations for the damage end states of Si and Ge recoils are plotted in Figs. 2 and 3, respectively.

**Table 1:** The calculated threshold displacement energies and associated defects after the recoil events in bulk Si and Ge. V_*X*_: *X* vacancy (*X* = Si or Ge); *X*_int_: *X* interstitial (*X* = Si or Ge)

	Bulk Si		Bulk Ge	
Direction	E_d_ (eV)	Defect type	E_d_ (eV)	Defect type
[001]	20, 17.4^a^, 21^c^	V_Si_ + Si_int_	18, 18.5^b^, ~ 18^f^	V_Ge_ + Ge_int_
[110]	47, 24^a^, ~ 47.6^d^	2V_Si_ + 2Si_int_	28.5	V_Ge_ + Ge_int_
[111]	9.5, 11.3^a^, ~ 12.9^e^	V_Si_ + Si_int_	9.5, 12.5^b^, ~ 15^c^	V_Ge_ + Ge_int_
$\left[\overline{1}\overline{1}\overline{1}\right]$	10	V_Si_ + Si_int_	9.5, 10.5^b^	V_Ge_ + Ge_int_

^a^Ref. [17]

^b^Ref. [20]

^c^Ref. [32]

^d^Ref. [33]

^e^Ref. [34]

^f^Ref. [36]

For bulk Si, the *E*_d_ values are slightly smaller than the experimental results of 21 eV for [001] [32], ~ 47.6 eV for [110] [33], and ~ 12.9 eV for [111] [34] directions, and both the experiment and our calculations reveal that the damaged end states are Frenkel pair (FP) defect. It is also noted that *E*_d_ values in the present study are generally comparable with the MD results reported by Windl et al. [18], except the case of [110], for which our calculated value of 47 eV is much larger than the MD result of 24 eV. Previous AIMD simulation of ion-solid interactions in SiC revealed that the displacement event is actually a charge-transfer process and the charge transfer to and from recoiling atoms can alter the energy barriers and dynamics for stable defect formation [35]. The lower values of *E*_d_ found by AIMD compared to those determined by classical MD may be due to the fact that charge transfer that occurs during the recoil events is taken into account by the AIMD method, while in the classical MD simulation, the charge of atoms is fixed. In the study of Windl et al., the kinetic energy is transferred to the PKA to generate one silicon vacancy (V_Si_) and one silicon interstitial (Si_int_) defects [18]. By contrast, in our study, the associated defects for Si[110] displacement event contain two V_Si_ and two Si_int_ defects, resulting in higher energies for the formation of the damaged states. The *E*_d_ values for Si[111] and Si$\left[\overline{1}\overline{1}\overline{1}\right]$ are very close to each other, i.e., 9.5 and 10 eV, respectively. In both cases, the created defects are V_Si_ and Si_int_ (see Fig. 2c, d), whereas the mechanisms of defect generation show different character. In the case of Si[111], the Si PKA moves along the $\left[11\overline{1}\right]$ direction due to the repulsive interactions and collides with its neighboring Si atom. The Si PKA then scatters away to occupy an interstitial site (Si_int_), and the replaced Si moves back to the lattice site of PKA. The associated defects are one V_Si_ and one Si_int_ defects. As for Si$\left[\overline{1}\overline{1}\overline{1}\right]$, the displacement event is relatively simpler, i.e., the Si PKA moves 4.69 Å away from its lattice site to form a Si_int_ defect. In the cases of Si[001] and Si[110], the E_d_s are determined to be 20 and 47 eV, respectively, indicating that the Si atoms are more difficult to be displaced along the [110] direction. The damage end states for Si[001] and Si[110] are somewhat different. In the case of Si[001], the PKA receives kinetic energy and moves along the [001] direction to collide with its neighboring atoms. The replaced Si atom keeps moving and occupies an interstitial site, as shown in Fig. 2a. As for Si[110], the PKA scatters toward the$\left[11\overline{1}\right]$direction due to the repulsive interactions between the PKA and its neighboring atoms and hits one neighboring Si atom (Si1). Then, the Si PKA rebounds toward the [111] direction to replace another Si atom (Si2), and the Si2 atom occupies an interstitial site in the end. The Si1 atom receives sufficient energy to move along the [110] direction and replaces its neighboring Si atom (Si3), which forms an interstitial defect. In the end, the associated defects are two V_Si_ and two Si_int_ defects, as shown in Fig. 2b.

For bulk Ge, the values of *E*_d_ are in good agreement with the experimental value of ~ 18 eV [36] and the theoretical value of 18.5 eV [20] for [001] direction. It is noted that the present value of 9.5 eV is comparable to the Holmström’s result of 12.5 eV [20] for [111] direction, which are smaller than the experimental value of ~ 15 eV [36]. For Ge[111] and Ge$\left[\overline{1}\overline{1}\overline{1}\right]$, the determined *E*_d_ values are as small as 9.5 eV, indicating that the Ge atoms are easily to be displaced along these two directions. In both cases, the associated defects are germanium vacancy and germanium interstitial (see Fig. 3c, d). For Ge$\left[\overline{1}\overline{1}\overline{1}\right]$, the Ge PKA does not follow a straight path, but gets strongly deflected by one of its nearest neighbors to occupy an interstitial site (Ge_int_). By contrast, in the case of Ge[111], the Ge PKA moves 4.92 Å along the [111] direction to form an interstitial defect (Ge_int_). As compared with the *E*_d_ of Ge[001], the value of Ge[110] is 10 eV larger, indicating that the Ge atom is more difficult to be displaced along the [110] direction. Although the associated defects for Ge[001] and Ge[110] are similar, the mechanisms for defect generation are somewhat different. The Ge PKA receives kinetic energy and moves along the [001] direction to collide with its neighboring atoms. The replaced Ge atom keeps moving and occupies an interstitial site, as shown in Fig. 3a. As for Ge[110], the Ge recoil collides with its first neighboring Ge atom (Ge1) along the [110] direction and rebounds along the [111] direction, resulting in the formation of Ge_int_. The Ge1 atom leaves its lattice site and replaces its neighboring Ge atom (Ge2). Subsequently, the Ge2 atom moves back to the lattice site of Ge1 and eventually only one V_Ge_ and one Ge_int_ defects are formed, as shown in Fig. 3b. These results suggest that in bulk Si and Ge, the E_d_s are strongly dependent on the crystallographic direction, and the atoms are more difficult to be displaced along the [110] direction. The radiation damage end states in bulk Si and Ge are mainly FP defects, i.e., vacancy and interstitial defects.

### The Displacement Events in Si/Ge Superlattice

**Fig. 4:**
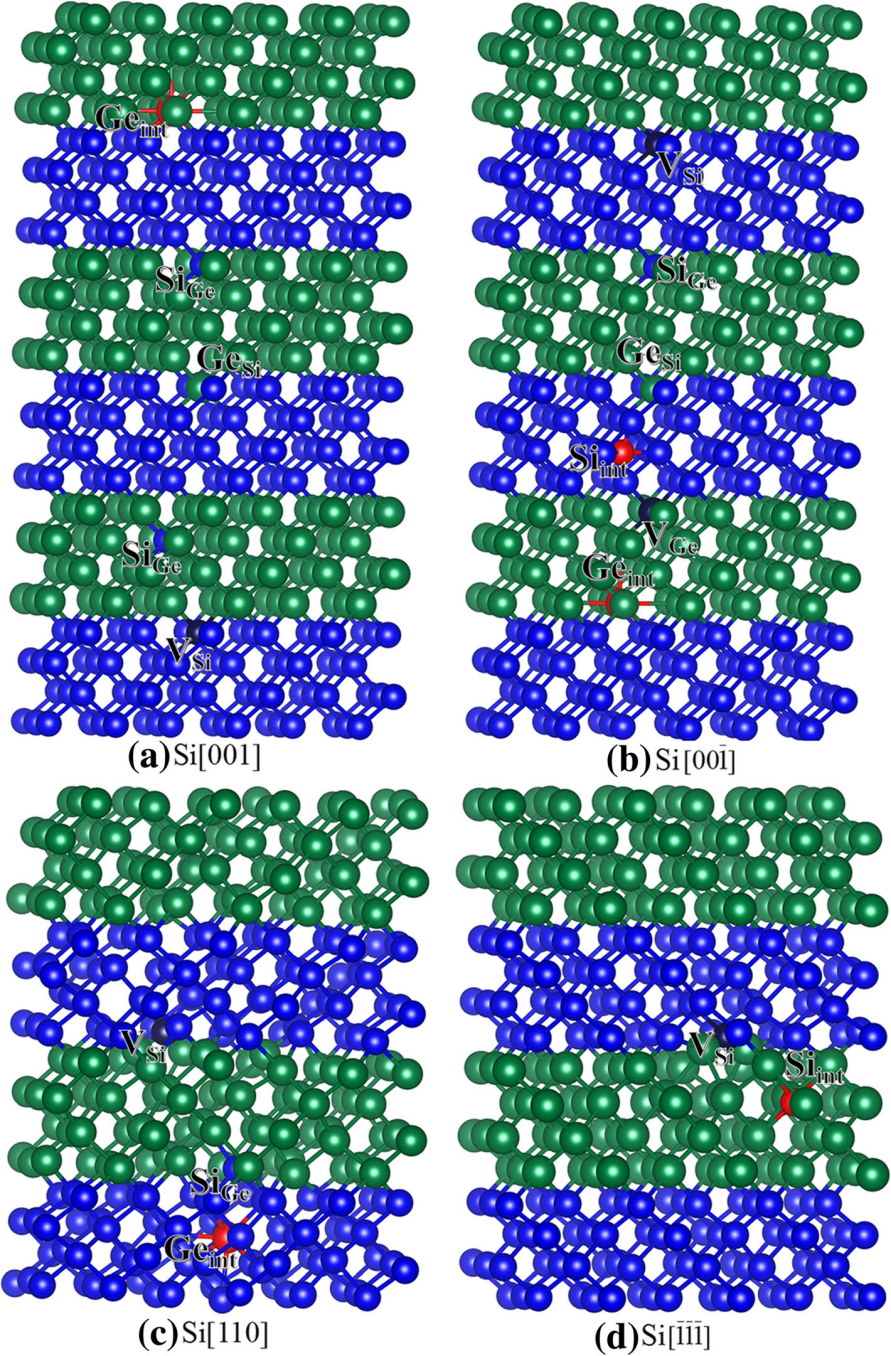
**a**–**d** Schematic view of geometrical structures of damage Si/Ge superlattice after Si recoil events. The blue and green spheres represent the Si and Ge atoms, respectively. V_*X*_: *X* vacancy (*X* = Si or Ge); *X*_int_: *X* interstitial (*X* = Si or Ge); *X*_*Y*_: *X* occupying the *Y* lattice site (*X* and *Y* = Si or Ge). The purple and red spheres represent the vacancy and interstitial defects, respectively

**Fig. 5:**
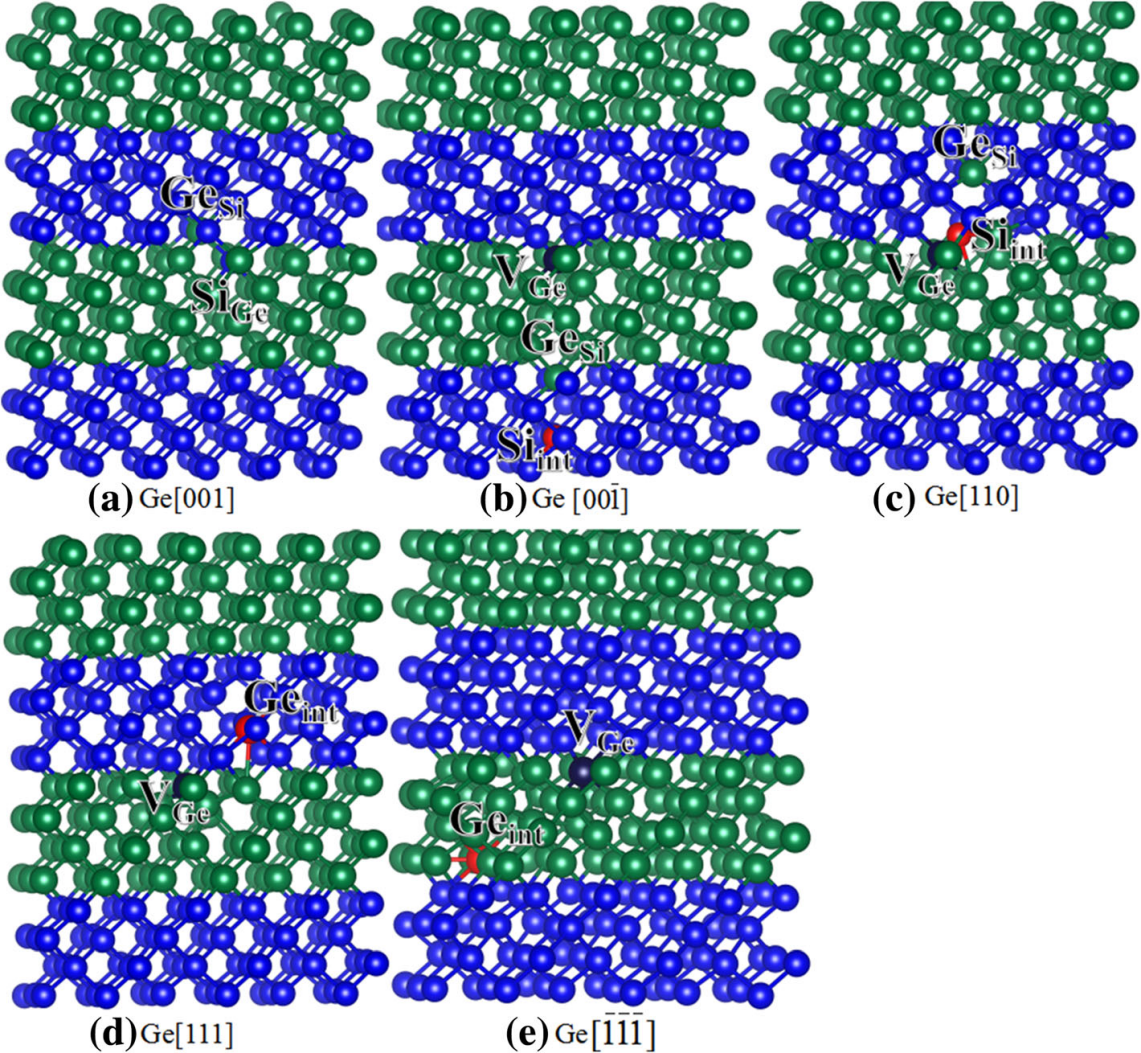
**a**–**e** Schematic view of geometrical structures of damage Si/Ge superlattice after Ge recoil events. The blue and green spheres represent the Si and Ge atoms, respectively. V_*X*_: *X* vacancy (*X* = Si or Ge); *X*_int_: *X* interstitial (*X* = Si or Ge); *X*_*Y*_: *X* occupying the *Y* lattice site (*X* and *Y* = Si or Ge). The purple and red spheres represent the vacancy and interstitial defects, respectively

In this study, the displacement events of Si_2_/Ge_2_ SL, which contains two layers of Si alternating with two layers of Ge (see Fig. 1b), are considered. The Si and Ge atoms that are adjacent to the Si/Ge interface are selected as the PKA. The E_d_s for Si and Ge recoils and the associated defects are listed in Table 2. The defect configurations for Si and Ge recoils are illustrated in Figs. 4 and 5, respectively. It is noted that in the case of Si[111], no defects are created even at energies up to 100 eV. Due to the computational restrictions, we did not perform further simulations of recoil events at energies higher than 100 eV, and the exact *E*_d_ value for Si[111] is not determined.

**Table 2:** The calculated threshold displacement energies and associated defects after the recoil events in Si/Ge superlattice. V_*X*_: *X* vacancy (*X* = Si or Ge); *X*_int_: *X* interstitial (*X* = Si or Ge); *X*_*Y*_: *X* occupying the *Y* lattice site (*X* and *Y* = Si or Ge)

	Si recoils		Ge recoils	
Direction	E_d_ (eV)	Defect type	E_d_ (eV)	Defect type
[001]	46.5	V_Si_ + Si_Ge_ + Ge_Si_ + Si_Ge_ + Ge_int_	16	Si_Ge_ + Ge_Si_
$\left[00\overline{1}\right]$	42.5	V_Si_ + Si_Ge_ + Ge_Si_ + Si_int_ + Ge_int_ + V_Ge_	17.5	V_Ge_ + Ge_Si_ + Si_int_
[110]	38.5	V_Si_ + Si_Ge_ + Ge_int_	20	V_Ge_ + Ge_Si_ + Si_int_
[111]	> 100	–	10	V_Ge_ + Ge_int_
$\left[\overline{1}\overline{1}\overline{1}\right]$	10	V_Si_ + Si_int_	13.5	V_Ge_ + Ge_int_

In the Si/Ge SL structure, the Si PKA is found to be easily displaced along the $\left[\overline{1}\overline{1}\overline{1}\right]$ direction, as indicated by the small *E*_d_ value of 10 eV. The pathway for defect generation is very simple, i.e., the Si PKA moves 4.61 Å away from its lattice site and forms a Si_int_ defect. For Si[001] and Si$\left[00\overline{1}\right]$, the E_d_s are determined to be 46.5 and 42.5 eV, respectively, and the damaged defects are different as expected. In the case of Si[001], the Si PKA moves along the [001] direction to replace its neighboring Ge atom (Si_Ge_), and the replaced Ge atom collides with its adjacent Si atom and occupies its lattice site, forming a Ge_Si_ antisite defect. The replaced Si atom receives sufficient energy and further replaces another Ge atom (Si_Ge_), which finally occupies an interstitial site. Eventually, the associated defects are one V_Si_, one Ge_int_, and three antisite defects. As for Si$\left[00\overline{1}\right]$, two neighboring Ge atoms and one neighboring Si atom are also involved in the displacement event, and the damaged states contain two vacancies, two interstitials, and two antisite defects, as shown in Fig. 4b. In the case of Si [110], the Si atom moves to hit its neighboring Si atom and scatters toward the $\left[11\overline{1}\right]$ direction. Then, the Si PKA replaces one neighboring Ge atom, which occupies an interstitial site in the end. After the displacement events, the associated defects contain one V_Si_, one Si_Ge,_ and one Ge_int_ defects. As compared with the bulk Si, the Si atoms in Si/Ge SL are generally more difficult to be displaced except the case of [110] and the mechanisms of defect generation are more complex, indicating that the bulk Si and Si/Ge SL show different radiation responses to irradiation. Our results are consistent with the experiments carried out by Fonseca et al. and Leitão et al. [13, 14], who also found that the radiation resistance of the SL structure was enhanced as compared with the bulk silicon.

For Ge recoils in Si/Ge SL, the Ge atoms are easily to be displaced along the [111] and $\left[\overline{1}\overline{1}\overline{1}\right]$ directions, which are similar to the Ge recoil events in bulk Ge. Although the radiation damage end states for Ge[111] and Ge$\left[\overline{1}\overline{1}\overline{1}\right]$ are very similar, i.e., Ge FP defects, the mechanisms of defect generation are different. In the case of Ge[111], the Ge PKA moves 4.77 Å away from its lattice site and forms a Ge_int_ defect. For the Ge$\left[\overline{1}\overline{1}\overline{1}\right]$, the Ge atom moves along the $\left[\overline{1}\overline{1}\overline{1}\right]$ direction to replace its neighboring Ge atom. The collided Ge atom moves along this direction and occupies an interstitial site in the end. It is noted that the E_d_ values of 16 eV for Ge[001] and 17.5 eV for Ge$\left[00\overline{1}\right]$ are comparable with the value of 18 eV for Ge[001] in bulk Ge, whereas the associated defects show different character. In the case of Ge[001], the Ge PKA receives sufficient energy but scatters along the [111] direction to replace its neighboring Si atom, forming a Ge_Si_ antisite defect. Then, the replaced Si atom occupies the Ge PKA lattice site and forms an antisite defect (Si_Ge_). In the case of Ge$\left[00\overline{1}\right]$, the Ge PKA moves 5.63 Å away to replace its neighboring Si atom. The Si atom moves along this direction and forms a Si_int_ defect. As compared with the Ge[110] in bulk Ge, the E_d_ for Ge[110] in Si/Ge SL is 8.5 eV smaller, and the associated defects are more complex, as indicated by one V_Ge_, one Ge_Si_, and one Si_int_ defects. Comparing the Ge recoil events in bulk Ge and SL, we find that the Ge atoms in Si/Ge SL are more resistant along the [110] direction. For other displacement events, the E_d_s are generally comparable with those for bulk states. However, the radiation damage end states in bulk Ge and Si/Ge SL are different, and some antisite defects are created in Si/Ge SL structure. These results suggest that the Ge recoils in Si/Ge SL structure show different radiation responses to irradiation. Comparing the Si and Ge recoils in SL structure, we find that the displacement events of Si atoms are much more affected than Ge, i.e., the E_d_s for Si atoms in SL structure are generally increased, which may lead to enhanced radiation resistance of Si/Ge SL. Sobolev et al. have found that the Si/Ge SLs show extraordinarily high radiation hardness as compared with bulk Si [12], which is consistent with our results.

### The Defect Formation Energy and Migration Barrier in Bulk Si, Ge, and Si/Ge Superlattice

In bulk Si and Ge, the damaged states are mainly vacancy and interstitial defects. As for Si/Ge SL, the associated defects contain vacancy, interstitial, and antisite defects and the mechanisms of defect generation are generally more complex. The discrepancy in the resistance to defect formation between bulk component materials and Si/Ge SL may result in their different radiation tolerances. To further investigate the origin of the different radiation responses of these semiconductor materials, we calculate the formation energies of vacancy, interstitial and antisite defects in bulk states and SL structures and the migration barrier of the most favorable defects employing density functional theory method. The computations are based on a supercell consisting of 64 atoms, with a 6 × 6 × 6 k-point sampling in real space and a cutoff energy of 500 eV.

The defect formation energies in bulk Si, Ge, and Si/Ge SL are listed in Table 3, along with other calculated results. In bulk Si, the formation energies for V_Si_, Si_int_, and Si FP defects are calculated to be 3.60, 3.77, and 4.62 eV, respectively, which are in reasonable agreement with other calculations [37–40]. Our results indicate that the V_Si_ defect is easier to be created in bulk Si. Similarly, the V_Ge_ defect in bulk Ge is energetically more favorable than the Ge_int_ and Ge FP defects, as indicated by the smallest defect formation energy of 2.23 eV, which compares well with the theoretical value of 2.09 eV [39]. As for the Si/Ge SL, the formation energy of V_Ge_ is determined to be 2.73 eV, which is smaller than the formation energies of other defects. The next favorable defect is the V_Si_ defect, and the formation energy is determined to be 2.85 eV. It is noted that the value of 3.52 eV for Ge_int_ is smaller than the value of 3.77 eV for Si_int_ defect. As for FP defect, the formation energy is obviously larger, i.e., 5.19 eV for Si FP and 5.01 eV for Ge FP, suggesting that the FP defects are difficult to be created. As compared with the bulk states, the defect formation energies for Si/Ge SL structure are generally larger except for the defects of V_Si_ and Si_int_, indicating that in SL structure, the point defects are generally more difficult to form. Such discrepancy in the resistance to defect formation between bulk states and Si/Ge SL structure may result in their different responses to irradiation.

**Table 3:** The defect formation energies in bulk Si, Ge, and Si/Ge superlattice. V_*X*_: *X* vacancy (*X* = Si or Ge); *X*_int_: *X* interstitial (*X* = Si or Ge); FP defect: Frenkel pair defect

Defect type	Defect formation energies (eV)		
	Si/Ge SL	Bulk Ge	Bulk Si
V_Si_	2.85	–	3.60, 3.61^a^, 3.56^b^
V_Ge_	2.73	2.23, 2.09^a^	–
Si_int_	3.77	–	3.77, 3.75^c^,3.29^d^
Ge_int_	3.52	2.97, 2.92^e^	–
Si FP	5.19	–	4.62, 4.26^b^
Ge FP	5.01	4.15	–

^a^Ref. [39]

^b^Ref. [38]

^c^Ref. [40]

^d^Ref. [37]

^e^Ref. [42]

Based on the optimized structures, the migration behaviors of the V_Ge_ and V_Si_ defects that are the most favorable defects in bulk and Si/Ge SL structures are further investigated. The V_Ge_ and V_Si_ defects which are adjacent to the Si/Ge interface are taken into account, and the migration barriers are summarized in Table 4. It is noted that the migration barriers along the [100] and [110] directions for V_Ge_ defects are smaller than those for V_Si_ defects, and the energy barrier for V_Ge_ migration along the [111] direction is slightly larger than that for V_Si_ migration, which are consistent with the results reported by Cowern et al. [41].

**Table 4:** The defect migration barrier in bulk Si, Ge, and Si/Ge superlattice. V_*X*_: *X* vacancy (*X* = Si or Ge)

Defect type	Direction	Migration barrier (eV)		
		Si/Ge SL	Bulk Ge	Bulk Si
V_Si_	[100]	3.92	–	4.32
	[110]	2.14	–	2.12, 2.85^a^
	[111]	0.49	–	0.11
V_Ge_	[100]	2.87	3.67	–
	[110]	1.39	1.94, 2.1^a^	–
	[111]	0.61	0.14	–

^a^Ref. [41]

**Fig. 6:**
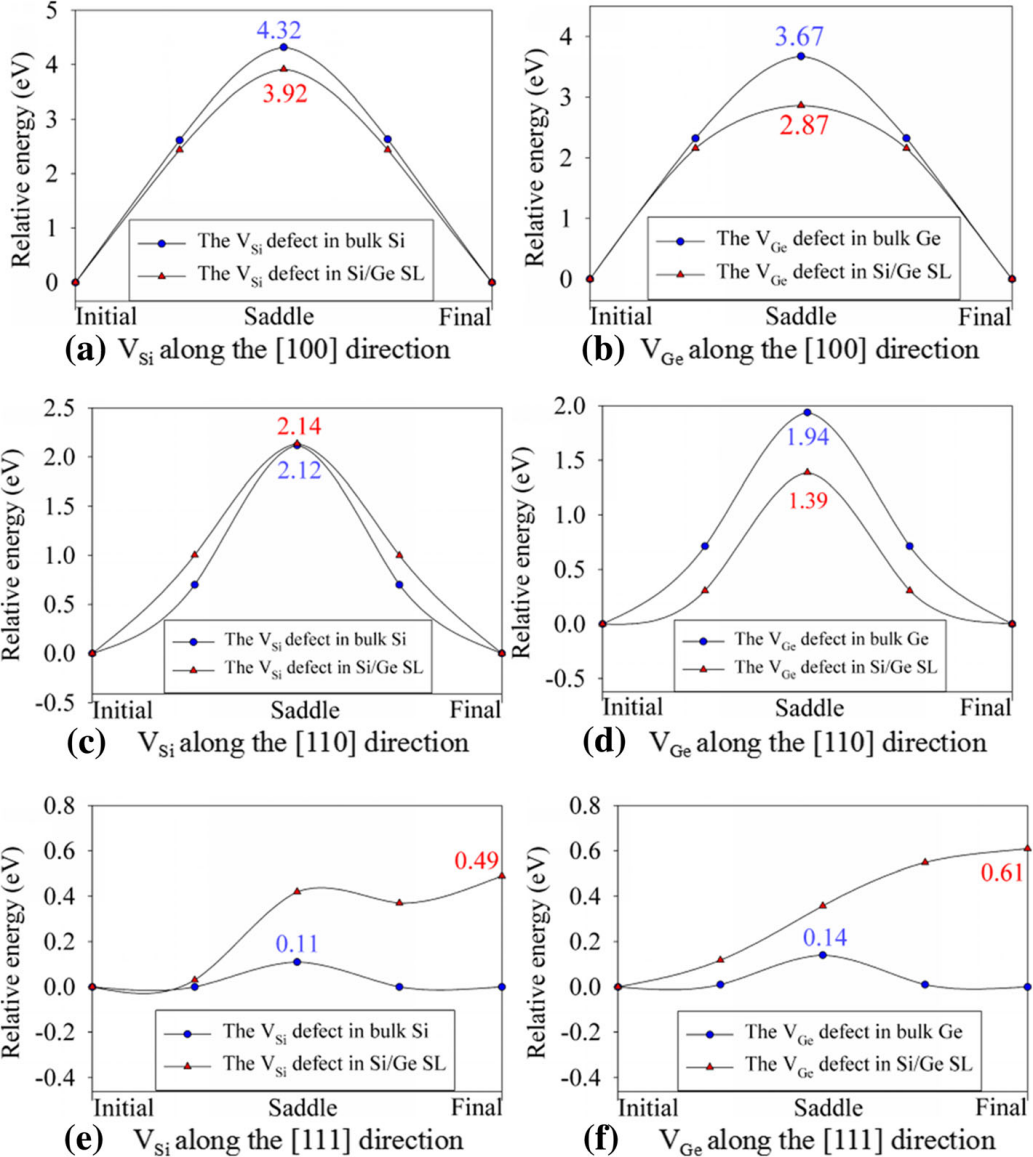
The migration barrier of silicon vacancy (V_Si_) and germanium vacancy (V_Ge_) defects obtained by a cluster nudged elastic band method. **a** V_Si_ along the [100] direction; **b** V_Ge_ along the [100] direction; **c** V_Si_ along the [110] direction; **d** V_Ge_ along the [110] direction; **e** V_Si_ along the [111] direction; **f** V_Ge_ along the [111] direction

The energy landscapes of defect migration along the [100], [110], and [111] directions are plotted in Fig. 6. In Fig. 6a, the migration barriers of the V_Si_ defect along the [100] direction are determined to be 4.32 and 3.92 eV in bulk Si and Si/Ge SL, respectively. As for the [110] direction, the migration barrier of 2.14 eV for V_Si_ in the Si/Ge SL structure is very close to the value of 2.12 eV in bulk Si. Comparing the migration barrier along each direction, we find that the [111] direction is the most favorable migration direction for both Si and Ge vacancies, as indicated by the significantly smaller migration barriers. Especially, the V_Si_ defects migrate more easily along the [111] direction in bulk Si than Si/Ge SL, since the energy barrier of 0.11 eV in the bulk state is much smaller (see Fig. 6e). As for the V_Ge_ defects, the migration barriers along the [100] direction are calculated to be 3.67 eV in bulk Ge and 2.87 eV in Si/Ge SL. In the case of [110] direction, the energy barriers are determined to be 1.94 and 1.39 eV in the bulk and SL structures, respectively. Similar to the case of Si vacancy migration, the V_Ge_ defects are easier to migrate along the [111] direction. Also, the migration occurs more easily in bulk Ge than Si/Ge SL, as shown in Fig. 6f. Our calculations suggest that both Si and Ge vacancies are more mobile in the bulk states than SL structure, which may result in void formation and even volume swelling. This may contribute to different responses to irradiation for the bulk and SL structures.

## Conclusions

In summary, low-energy displacement events in bulk Si, Ge, and Si/Ge superlattice (SL) have been investigated by an ab initio molecular dynamics method. In bulk Si and Ge, the threshold displacement energies are shown to be dependent on the crystallographic direction and the atoms are more difficult to be displaced along the [110] direction. The damaged states in bulk states are mainly vacancy and interstitial defects. In the Si/Ge SL structure, the Si atoms are more resistant along the [111] direction, while the Ge atoms are more difficult to be displaced along the [110] direction. Our calculations show that the energies for the Ge recoils in the SL structure are generally comparable to those in the bulk Ge, whereas the energies for the Si recoils in the SL structure are generally much larger than those in bulk Si, indicative of enhanced radiation resistance of the Si/Ge SL. Defect formation energy calculations show that the point defects in the Si/Ge SL generally have higher formation energies, indicating that in the SL structure the point defects are generally more difficult to form. It is also found that the [111] direction is the most favorable migration path for both Si and Ge vacancies, and both vacancies are more mobile in the bulk states than in SL structure. Our calculations suggest that the enhanced radiation resistance of Si/Ge SL is beneficial to its application as electronic and optoelectronic devices under extreme working conditions like radiation.

## Abbreviations

AIMD: Ab initio molecular dynamics
E_d_: Threshold displacement energy
FP: Frenkel pair
Ge: Germanium
Ge_int_: Germanium interstitial
Ge_Si_: Germanium occupying the silicon lattice site
LDA: Local-density approximation
MD: Molecular dynamics
NVE: Microcanonical ensemble
PKA: Primary knock-on atom
PL: Photoluminescence
QD: Quantum dot
QW: Quantum well
Si: Silicon
SIESTA: Spanish Initiative for Electronic Simulations with Thousands of Atoms
Si_Ge_: Silicon occupying the germanium lattice site
Si_int_: Silicon interstitial
SL: Superlattice
SZP: Single-ζ basis sets plus polarization orbital
V_Ge_: Germanium vacancy
V_Si_: Silicon vacancy
